# Comparative Genomic Analysis of *Bacillus amyloliquefaciens* and *Bacillus subtilis* Reveals Evolutional Traits for Adaptation to Plant-Associated Habitats

**DOI:** 10.3389/fmicb.2016.02039

**Published:** 2016-12-20

**Authors:** Nan Zhang, Dongqing Yang, Joshua R. A. Kendall, Rainer Borriss, Irina S. Druzhinina, Christian P. Kubicek, Qirong Shen, Ruifu Zhang

**Affiliations:** ^1^Jiangsu Key Lab and Engineering Center for Solid Organic Waste Utilization, National Engineering Research Center for Organic-based Fertilizers, Jiangsu Collaborative Innovation Center for Solid Organic Waste Resource Utilization, Nanjing Agricultural UniversityNanjing, China; ^2^Department of Science and Technology, Evangel UniversitySpringfield, IL, USA; ^3^Fachgebiet Phytomedizin, Institut für Agrar- und Gartenbauwissenschaften, Humboldt- Universität zu BerlinGermany; ^4^Research Area Biotechnology and Microbiology, Institute of Chemical Engineering, Vienna University of TechnologyVienna, Austria; ^5^Key Laboratory of Microbial Resources Collection and Preservation, Ministry of Agriculture, Institute of Agricultural Resources and Regional Planning, Chinese Academy of Agricultural SciencesBeijing, China

**Keywords:** *Bacillus subtilis* and *Bacillus amyloliquefaciens*, genome evolution, horizontal gene transfer, plant-associated, phylogenomics, rhizosphere adaptation

## Abstract

*Bacillus subtilis* and its sister species *B. amyloliquefaciens* comprise an evolutionary compact but physiologically versatile group of bacteria that includes strains isolated from diverse habitats. Many of these strains are used as plant growth-promoting rhizobacteria (PGPR) in agriculture and a plant-specialized subspecies of *B. amyloliquefaciens—B. amyloliquefaciens* subsp. *plantarum*, has recently been recognized, here we used 31 whole genomes [including two newly sequenced PGPR strains: *B. amyloliquefaciens* NJN-6 isolated from *Musa* sp. (banana) and *B. subtilis* HJ5 from *Gossypium* sp. (cotton)] to perform comparative analysis and investigate the genomic characteristics and evolution traits of both species in different niches. Phylogenomic analysis indicated that strains isolated from plant-associated (PA) habitats could be distinguished from those from non-plant-associated (nPA) niches in both species. The core genomes of PA strains are more abundant in genes relevant to intermediary metabolism and secondary metabolites biosynthesis as compared with those of nPA strains, and they also possess additional specific genes involved in utilization of plant-derived substrates and synthesis of antibiotics. A further gene gain/loss analysis indicated that only a few of these specific genes (18/192 for *B. amyloliquefaciens* and 53/688 for *B. subtilis*) were acquired by PA strains at the initial divergence event, but most were obtained successively by different subgroups of PA stains during the evolutional process. This study demonstrated the genomic differences between PA and nPA *B. amyloliquefaciens* and *B. subtilis* from different niches and the involved evolutional traits, and has implications for screening of PGPR strains in agricultural production.

## Introduction

As a group of Gram-positive, aerobic, and endospore-forming *Firmicutes* bacteria, commonly known as the “*Bacillus subtilis* group” or *B. subtilis sensu lato* is of importance in both basic and applied microbiology (Fritze, 2004). Members of this group, *B. subtilis sensu stricto* and a recently distinguished sister species *B. amyloliquefaciens*, have been both widely used as producers of commercial chemicals in industry (Harwood, 1992; Geng et al., 2011), and beneficial agents for plant growth promotion and suppression of soil-borne diseases in agriculture (Chen et al., 2007, 2009; Fan et al., 2012).

*B. subtilis* and *B. amyloliquefaciens* are frequently isolated from various niches, including soil, animal feces, human food, aquatic environments, and so on (Earl et al., 2008). It is known that bacterial adaptation to different environments during the evolution process would lead to differentiation as indicated by different genomic and physiological characteristics (Earl et al., 2008; de Wit et al., 2012). For instance, *B. amyloliquefaciens* FZB42^T^ isolated from the rhizosphere of *Beta vulgaris* (sugar beet) is widely used as a commercial plant growth-promoting rhizobacteria (PGPR), based on its abilities for plant root-colonizing and antibiotics production (Borriss et al., 2011; Chowdhury et al., 2015); while *B. amyloliquefaciens* LL3, isolated from fermented vegetables (Korean bibimbap), has been applied as an industrial strain for poly-γ-glutamic acid production (Geng et al., 2011). Recently, whole-genome sequencing of numerous *Bacillus* strains has uncovered the molecular basis for their versatile performance under different environments (Kunst et al., 1997; Deng et al., 2011; Blom et al., 2012; He et al., 2012). Based on a comparative genomic analysis within *Bacillus* spp., Alcaraz et al. indicated that the pathogen *B. cereus* possesses enriched genes involved in defense mechanisms, while the soil dweller *B. subtilis* harbors over-represented genes relevant to carbohydrates degradation (Alcaraz et al., 2010). Recently, it was proposed that *B. amyloliquefaciens* comprises two subspecies: The plant-associated *B. amyloliquefaciens* subsp. *plantarum*, and the non-plant-associated *B. amyloliquefaciens* subsp. *amyloliquefaciens*, based on both phylogenic analysis and physiological characteristics such as abilities for root colonization and production of plant growth hormones/antibiotics (Borriss et al., 2011); however, the evolution events leading to such divergence has not yet been investigated in more detail. In addition, for *B. subtilis* it is unclear whether they can be distinguished by different characteristics typical for plant-associated and non-plant-associated life style (Yi et al., 2014).

Based on this background, for better understanding of the genomic differences between *B. subtilis* and *B. amyloliquefaciens* strains isolated from plant-associated niches (rhizosphere) and non-plant-associated niches, we try to answer three questions: (i) Are there any significant genomic differences between plant-associated (PA) *B. amyloliquefaciens/B. subtilis* strains and non-plant-associated (nPA) strains? (ii) If yes, what are the specific genes caused the differences and potentially involved in rhizosphere adaptation? (iii) And, what's the evolution process of these specific genes, i.e., did the specific genes were absent in the ancestor but were acquired by the PA strains during the history (simultaneously or continuously), or they were shared by all strains but were discarded by the nPA strains? In this study, 29 previously published *B. amyloliquefaciens* and *B. subtilis* genomes, and two newly sequenced rhizospheric strains: *B*. *amyloliquefaciens* NJN-6 isolated from *Musa* sp. (banana) and *B. subtilis* HJ5 from *Gossypium* sp. (cotton) were collected to perform the comparative genomic and dynamic gene gain/loss analysis. Our results revealed genomic differences between PA and nPA *B. amyloliquefaciens/B. subtilis*, and indicated that the PA strains possess additional genes involved in utilization of plant-derived substrates and synthesis of antibiotics, which have arisen via horizontal gene transfer (HGT) events during the evolutionary process.

## Results

### Genome structure of two newly isolated rhizosphere strains of *Bacillus* spp.

The whole genome sequencing of two newly isolated rhizospheric PGPR strains, *B. amyloliquefaciens* NJN-6 and *B. subtilis* HJ5, was performed as described in Materials and Methods. The genome sizes of NJN-6 and HJ5 were found to be similar (4.05 and 4.01 Mb, respectively; Table 1). The number of predicted protein-coding genes of NJN-6 and HJ5 were 3894 and 3917, respectively (Table 1). The annotated sequences of NJN-6 and HJ5 were submitted to the NCBI GenBank database with the accession number of CP007165 and CP007173, respectively.

**Table 1:** **Summary statistics and information on the genome and the isolation of the 31 *Bacillus* genomes in this study**.

**Species**	**Subspecies^a^**	**Strain**	**Genome size (Mbp)**	**16S rRNA operons**	**Substrate**	**Country**	**Reference**	**NCBI Accession No**.
*B. amyloliquefaciens*	*plantarum*	NJN-6	4.05	8	*Musa* sp. rhizosphere	China	This study	NZ_CP007165/CP007165
		LFB112	3.94	9	Chinese herbs		Cai et al., 2014	NC_023073/CP006952
		Y2^b^	4.24	10	*Triticum* spp. rhizosphere		He et al., 2012	NC_017912/CP003332
		CAU B946	4.02	10	*Oryza sativa* rhizosphere		Blom et al., 2012	NC_016784/HE617159
		NAU-B3	4.2	11	*Triticum* spp. rhizosphere		Wu et al., 2013	NC_022530/HG514499
		YAU B9601-Y2^b^	4.24	10	*Triticum* spp. rhizosphere		He et al., 2012	NC_017061/HE774679
		SQR9	4.12	7	*Cucumis sativus* rhizosphere		Zhang et al., 2015	NZ_CP006890/CP006890
		CC178	3.92	9	*Cucumis sativus* phyllosphere	Korea	Kim et al., 2015	NC_022653/CP006845
		AS 43.3	3.96	10	*Triticum* spp. head	USA	Dunlap et al., 2013	NC_019842/CP003838
		UCMB5033	4.07	10	*Gossypium* sp.	Tajikistan	Niazi et al., 2014	NC_022075/HG328253
		UCMB5036	3.91	9	*Gossypium* sp. endophyte		Manzoor et al., 2013	NC_020410/HF563562
		UCMB5113	3.89	9	Soil	Ukraine	Niazi et al., 2014	NC_022081/HG328254
		FZB42^T^	3.92	10	Sugar beet field	Germany	Chen et al., 2007	NC_009725/CP000560
	n.a.	IT-45	3.93	10	n.a.	n.a.	CP004065	NC_020272/CP004065
	*amyloliquefaciens*	DSM7^T^	3.98	10	n.a.	Germany	Rückert et al., 2011	NC_014551/FN597644
		LL3	4	7	Korean bibimbap	Korea	Geng et al., 2011	NC_017190/CP002634
		TA208	3.94	7	Soil	China	Zhang et al., 2011	NC_017188/CP002627
		XH7	3.94	7	n.a.		Yang et al., 2011	NC_017191/CP002927
*B. subtilis*	n.a.	HJ5	4.01	7	*Gossypium* sp. rhizosphere	China	This study	NZ_CP007173/CP007173
	*subtilis*	BAB-1	4.02	11	*Gossypium* sp. rhizosphere		Guo et al., 2014	NC_020832/CP004405
	n.a.	XF-1	4.06	10	*Brassica rapa* rhizosphere		Guo et al., 2013	NC_020244/CP004019
	n.a.	BSn5	4.09	11	*Amorphophallus konjac* tissue		Deng et al., 2011	NC_014976/CP002468
	*subtilis*	168	4.22	10	n.a	USA	Kunst et al., 1997	NC_000964/AL009126
	*subtilis*	ATCC 6051	4.22	10		n.a.	Kabisch et al., 2013	NC_020507/CP003329
	n.a.	PY79	4.03	10		n.a.	Schroeder and Simmons, 2013	NC_022898/CP006881
	*subtilis*	RO-NN-1	4.01	10		Desert soil	Earl et al., 2012	NC_017195/CP002906
	*subtilis*	BSP1	4.04	10	Poultry	Switzerland	Schyns et al., 2013	NC_019896/CP003695
	n.a.	QB928	4.15	10	n.a.	n.a.	Yu et al., 2012	NC_018520/CP003783
	*natto*	BEST195	4.09	10	Miyagino-based natto	Japan	Kamada et al., 2014	NC_017196
	*spizizenii*	TU-B-10^T^	4.21	10	Soil	Tunisia	Earl et al., 2012	NC_016047/CP002905
		W23	4.03	8	n.a.	n.a.	Zeigler, 2011	NC_014479/CP002183

n.a. not available

a *Based on the information from NCBI (http://www.ncbi.nlm.nih.gov)*.

b *These two strains are exactly the same strain which have been independent sequenced and annotated by different laboratories*.

### The core- and pan-genomes of *B. amyloliquefaciens* and *B. subtilis*

The global gene repertoire of *B. amyloliquefaciens* and *B. subtilis* was determined by comparing the 31 genomes (Table 1). This resulted in a total of 123,555 genes, of which 121,056 (~97%) were clustered into 6596 orthologous groups (OGs) by OrthoMCL (Data Sheet S1). Of these OGs, 60 contained three or more gene copies, 279 contained two gene copies (doublets), and 6257 contained only a single gene copy (singletons).

**Figure 1:**
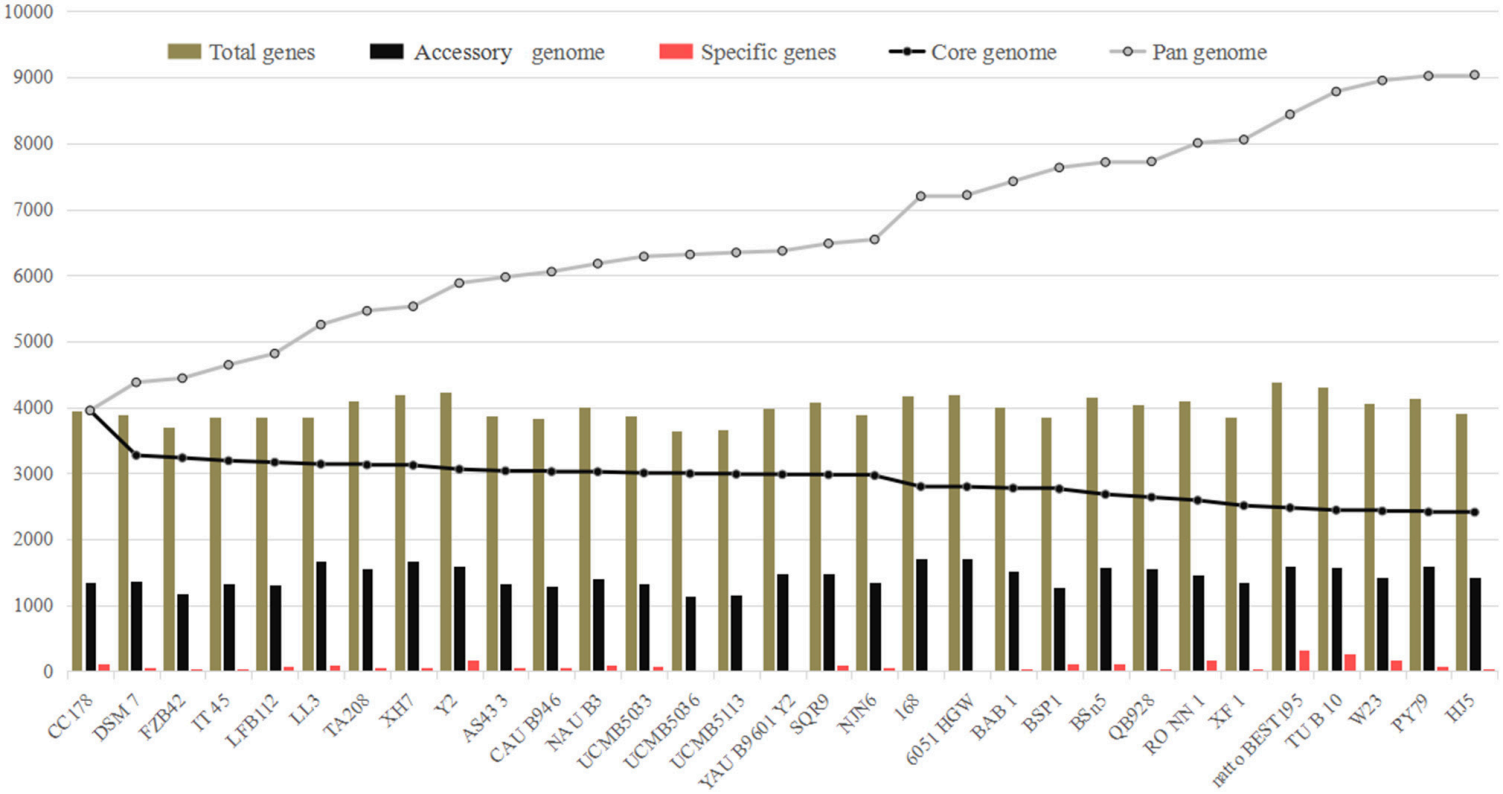
**Pan- and core-genome analysis of 18 *Bacillus amyloliquefaciens* and 13 *B*. *subtilis* strains**. The lines in gray and black represent the pan- and core-genomes, respectively. The pan-genome increased with addition of new strains to the study (9037 OGs), while the core genome decreased at a slow rate with added strains (2409).

The numbers corresponding to the core genome genes of *B. amyloliquefaciens* and *B. subtilis* respectively, decreased continually with the addition of new stains, while the pan-genome showed the opposite trend (Figure 1; Table S1). Both curves tended to be saturated when all 31 genomes were included. The *B. amyloliquefaciens* and *B. subtilis* core genomes consisted of 2409 OGs, representing around 60% of the average genome size (*N* = 31). The pan-genome for all lineages consisted of 9037 genes, corresponding to more than two-fold the average size of these 31 genomes (Figure 1).

### Phylogenomic and hierarchal clustering analysis demonstrated the differentiation between plant-associated (PA) and non-plant-associated (nPA) *B. amyloliquefaciens*/*B. subtilis*

**Figure 2:**
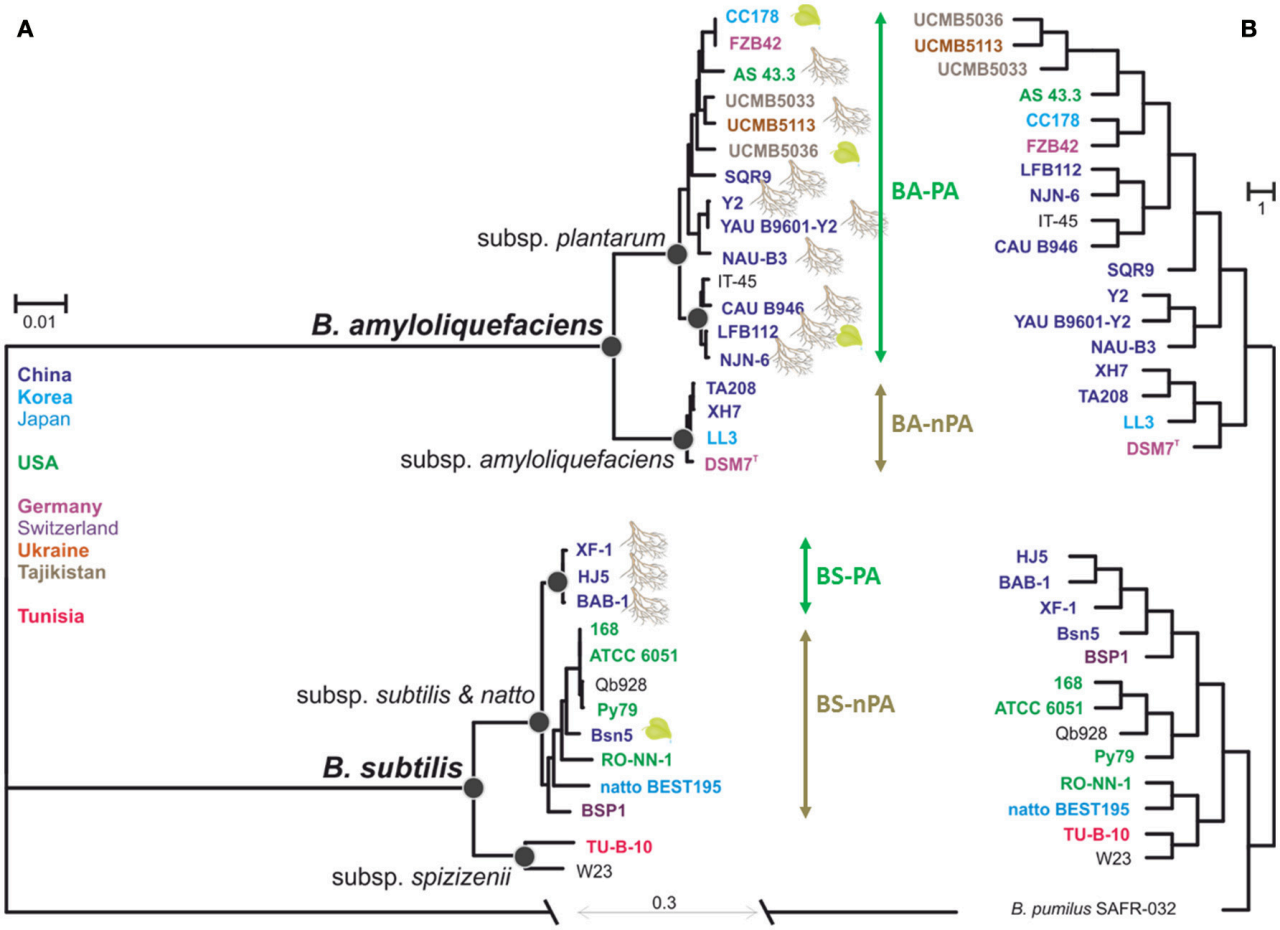
**Evolutionary relationship between the 31 Bacillus strains and hierarchal clustering**. **(A)** The maximum-likehood phylogeny derived from the alignment of 1835 concatenation core genes. Leaf clipart indicates association with plants. Colors indicate biogeographic origin of the strains. All nodes shown on the phylogram are supported by >85% bootstrap values and >0.94 posterior probabilities obtained after the 0.2 M mcmc generations in Bayesian analysis. Black circles indicate support for the most important nodes. **(B)** Hierarchal clustering among genomes using presence/absence of orthologous groups Almost all of nodes on the phylogram are supported by 97% >approximately unbiased *P*-values.

Phylogenetic relationship based on the core-genomes of *B. amyloliquefaciens* and *B. subtilis* was estimated from 1836 concatenated proteins, which resulted in a matrix of 500,457 amino acids residues. Figure 2A shows the consensus maximum likelihood phylogram rooted against *B. pumilus* SAFR-032 as an appropriate outgroup (Gioia et al., 2007). The resulting phylogenetic tree grouped the 31 genomes into two species clades with high bootstrap support values (Figure 2A) that supported the diversification of *B. subtilis sensu lato* into two monophyletic phylogenetic species, *B. subtilis* sensu stricto and *B. amyloliquefaciens*, respectively. Thirteen *B. amyloliquefaciens* strains that were isolated from plant-associated habitats (mainly rhizosphere) and IT-45, the one strain whose origin is unknown, but of which definition in NCBI organism overview has been reported as a PGPR strain (12th June 2013, http://www.ncbi.nlm.nih.gov/genome/848?genome_assembly_id=169345), formed a supported subclade corresponding to *B. amyloliquefaciens* subsp. *plantarum* (Figure 2A, plant-associated *B. amyloliquefaciens*, BA-PA group). Four strains that were isolated from none-plant-associated habitats formed another subclade corresponding to *B. amyloliquefaciens* subsp. *amyloliquefaciens* (non-plant-associated *B. amyloliquefaciens*, BA-nPA group). The phylogenetic structure of *B. subtilis sensu stricto* appeared to be more complex. Two strains, TU-B-10 and W23 formed a statistically supported basal subclade corresponding to the taxonomic definition of *B. subtilis* subsp. *spizizenii* (Zeigler, 2011), while the other eleven strains shared the same common ancestor and thus belonged to another subclade as *B. subtilis* subspecies *subtilis* (referred to *B. subtilis* in the rest of the article). Similar to another species, three Chinese rhizospheric strains being HJ5, BAB-1, and XF-1, formed an individual subclade (plant-associated *B. subtilis*, BS-PA). Interestingly this subclade did not include BSn5 strain isolated from calli tissue (*Amorphophallus konjac*) (Figure 2A). The remaining eight *B. subtilis* strains formed another clade defined as non-plant-associated *B. subtilis* group (BS-nPA). This phylogenetic structure indicates that within *B. subtilis*, rhizospheric strains share the same evolutionary pathway that was also observed for *B. amyloliquefaciens*.

In order to find out whether adaptation to the rhizosphere habitat went through convergent evolution in both species, we also looked at the genomic features of respective strains and compared them with saprotrophic members of each species (divergence of PA and nPA strains). For this reason we performed the hierarchal clustering analysis based on gene presence/absence among each strain and found it recovered most of the major groups found in the species tree (Figure 2B), which also clearly distinguished the PA and nPA groups within *B. amyloliquefaciens* and *B. subtilis*, respectively, only with some discrepancies.

### Core genome of plant-associated *B. amyloliquefaciens* and *B. subtilis* strains are more abundant in genes involved in metabolism of plant-derived substrates and synthesis of antibiotics

**Figure 3:**
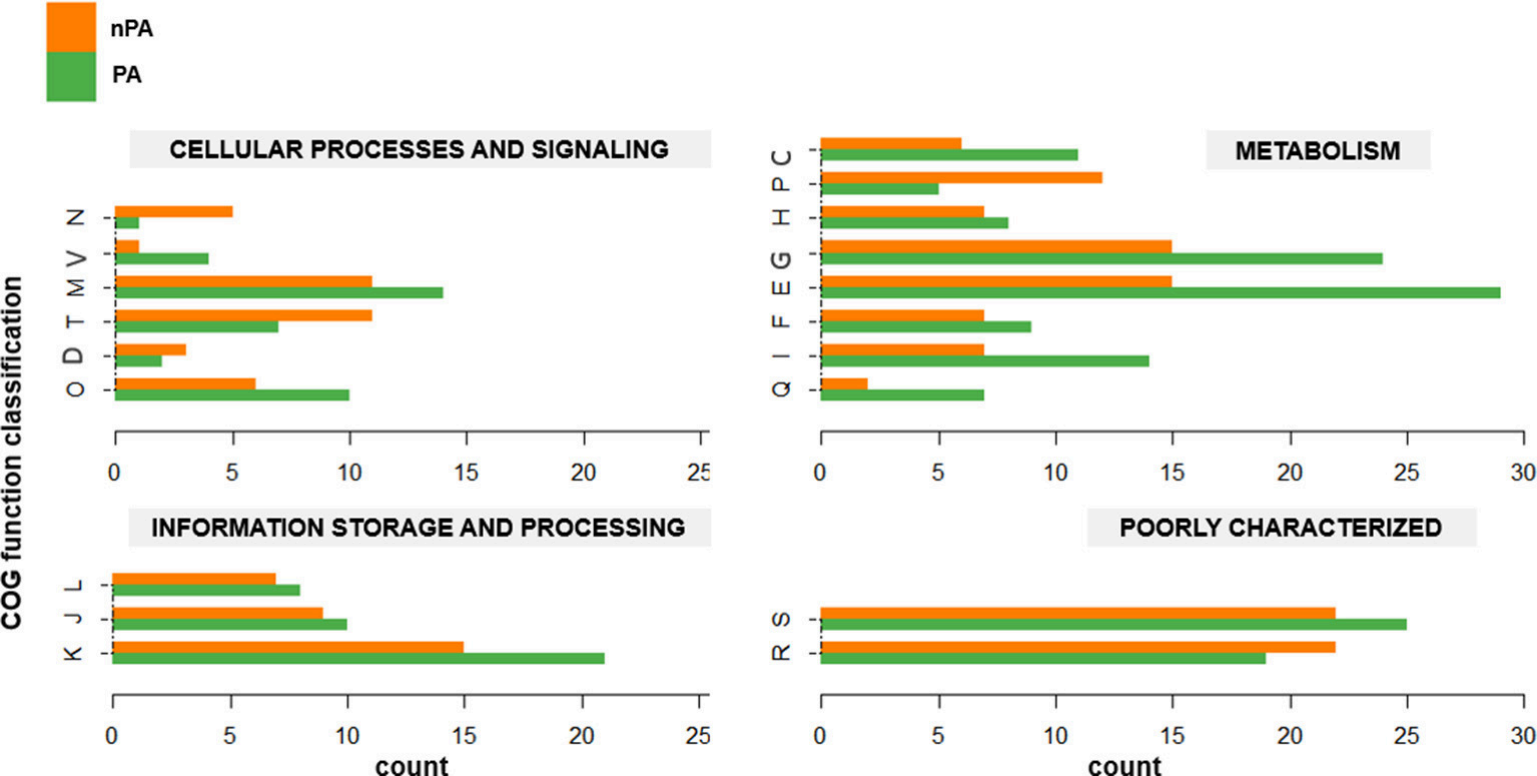
**Gene counts for each COG category in the core genome for non-plant-associated (nPA) vs. plant-associated (PA) groups**. Each pair of columns represents the COG category of the specific genes. Cellular process and signaling: D, cell cycle control, cell division, chromosome partitioning; M, cell wall/membrane/envelope biogenesis; N, cell motility; O, posttranslational modification, protein turnover, chaperones; T, signal transduction mechanisms; V, defense mechanisms. Metabolism: C, energy production and conversion; E, amino acid transport and metabolism; F, nucleotide transport and metabolism; G, carbohydrate transport and metabolism; H, coenzyme transport and metabolism; I, lipid transport and metabolism; P, inorganic ion transport and metabolism; Q, secondary metabolites biosynthesis, transport and catabolism. Information storage and processing: J, translation, ribosomal structure, and biogenesis; K, transcription; L, replication, recombination and repair. Poorly characterized: R, general function prediction only; S, function unknown.

The phylogenomic analysis (*vide supra*) revealed that in both *Bacillus* species, the plant-associated isolates formed their own clades. Subsequently, OrthoMCL was used to delimit the core genomes of PA and nPA strains respectively based on phylogenetic groups (Table S2). The core genome of the PA strains contained 2756 orthologous groups, while nPA strains contained 2714. Of these, 342 orthologous groups in the core genome of PA strains were identified not belong to core genome of the whole 31 strains, while there are 234 orthologous groups different for nPA (Tables S3, S4), which were defined as PA and nPA-specific core genome, respectively. It was found that 228 genes from PA-specific core genome and 183 from the nPA could be classified into multiple COGs. Among these, categories G (carbohydrate transport and metabolism) and E (amino acid transport and metabolism) were found to be more abundant in the PA-specific core genome as compared to that of nPA strains (24 vs. 15 for category G and 29 vs. 15 for E, respectively, Figure 3). In detail, the *bglC*/*eglS* (GP103033, encoding endo-1, 4-β-glucanase) and *bglS* (GP102126, endo-β-1,3-1,4 glucanase) involved in cellulose degradation, *xylA* (GP102236, xylose isomerase) and *xynB* (GP102238, β-xylosidase) involved in xylan degradation, *amyE* (GP103025, α-amylase) for saccharifying starch, and *pelB* (GP102863, pectate lyase) for pectate hydrolysis, were all identified in category G of the PA-specific core genome (Tables S3, S4). Category E mainly included genes encoding ABC-type amino acid transporters, such as *glnQHMP* (GP102791-94) for glutamine transport, and *opuCB* (GP102371) for glycine transport. Another two over-represented categories in the PA-specific core genome were Q (secondary metabolites biosynthesis, transport, and catabolism, 7 vs. 2 genes for PA and nPA, respectively) and I (lipid transport and metabolism, 14 vs. 7 genes, respectively) (Figure 3), which include genes involved in synthesis of polyketide antibiotics bacillaene (*bae* cluster, GP100000, 100027, and 102850-57) and difficidin (*dfnL*, GP100038) (Chen et al., 2009; Xu et al., 2014).

Interestingly, it was found that some sporulation genes were more frequently found among PA strains [e.g., *gerBA* (GP102918), *spoIIE* (GP102731), and *cotA* (GP102745), etc.; Table S3] while others more frequently among nPA strains [e.g., *gerPA* (GP102165), *spoIVB* (GP102275), and *cotJA* (GP102930), etc., Table S4]. This observation might be attributed to the complex and redundancy of sporulation genes (Tan and Ramamurthi, 2014), but the detailed comparison of sporulation abilities between PA and nPA strains, as well as the ecological significance in the rhizosphere, still need further exploration.

Since the limitation of core genome comparison (e.g., genes identified in PA-specific core-genome is probably because they are missing in one or a few nPA strains, but actually they are still encoded by many nPA strains), we further identified the genes that are present in all fourteen BA-PA strains but absent in all four BA-nPA strains (BA-PA specific genes), and those that occur in all three BS-PA strains but are absent in all ten BS-nPA strains (BS-PA specific genes). The 56 genes that only existed in the fourteen BA-PA strains included genes belonging to the *dfn* (*dfnBCJKMXY*, GP103464, and GP103547-52) and *mln* clusters (*mlnH* and *mlnI*, GP103539 and GP103540, respectively), which participate in nonribosomal synthesis of difficidin and macrolactin, respectively (Chen et al., 2009). Importantly, *amyE* (GP103025, α-amylase) and *bglC*/*eglS* (GP103033, endo-β-1, 4-glucanase) were also occurred in the PA-specific core genome summarized above (Table S5). This finding is in agreement with a previous study (Borriss et al., 2011) and implicated the importance of utilization of plant-derived polysaccharides and antibiotics production in rhizosphere competition. Most of the BS-PA specific genes were annotated as hypothetical proteins and their relationship to rhizosphere adaptation can therefore not be predicted; only GP105349 shows similarity to putative methyl-accepting transducer for sensing specific ligands possibly governing chemotaxis behavior of the bacterial cell in the rhizosphere (Table S6).

### PA-preferential genes were acquired successively by different subgroups of PA strains through horizontal gene transfer (HGT)

**Figure 4:**
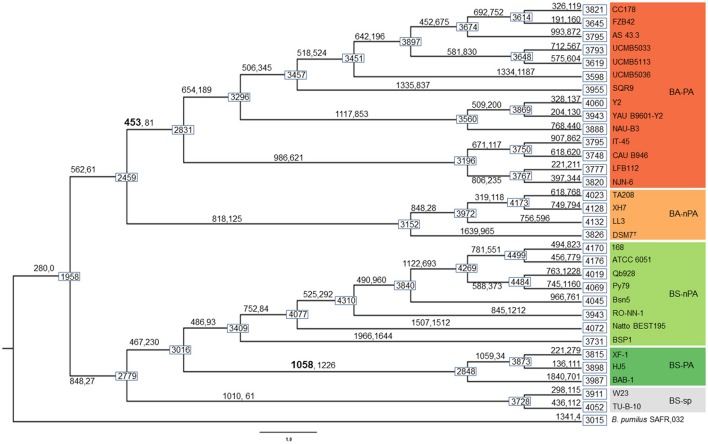
**Results of gene gain/loss analysis**. Boxes on nodes and tips of the phylogeny show genome size (genome size calculations exclude *one* of the orthologous groups that was excluded from the analysis, see Results). Numbers on branches indicate the number of genes gained (left, through HGT, gene duplication, or gene birth) and lost (right) at the divergence event.

Based on the comparative genomic analysis (*vide supra*), in order to further explore the dynamic events contributing to the differentiation between PA and nPA strains, AnGST was used to reconcile observed differences between gene and species phylogenetic trees (the phylogeny tree is same with that in Figure 2A) to identify the genomic events concomitant with diversification of both PA *Bacillus* clades (subsp.) during the evolutionary process. We detected 453 and 1058 genes that were gained on the branch leading to the last common ancestors of PA/nPA *B. amyloliquefaciens* and *B. subtilis*, respectively (Figure 4). Of these genes, 192 in the BA-PA and 688 in the BS-PA cluster were predicted to have been gained through HGT from other strains included in this analysis. The remaining genes were attributed to gene duplication and speciation (David and Alm, 2011) (Table S7).

**Figure 5:**
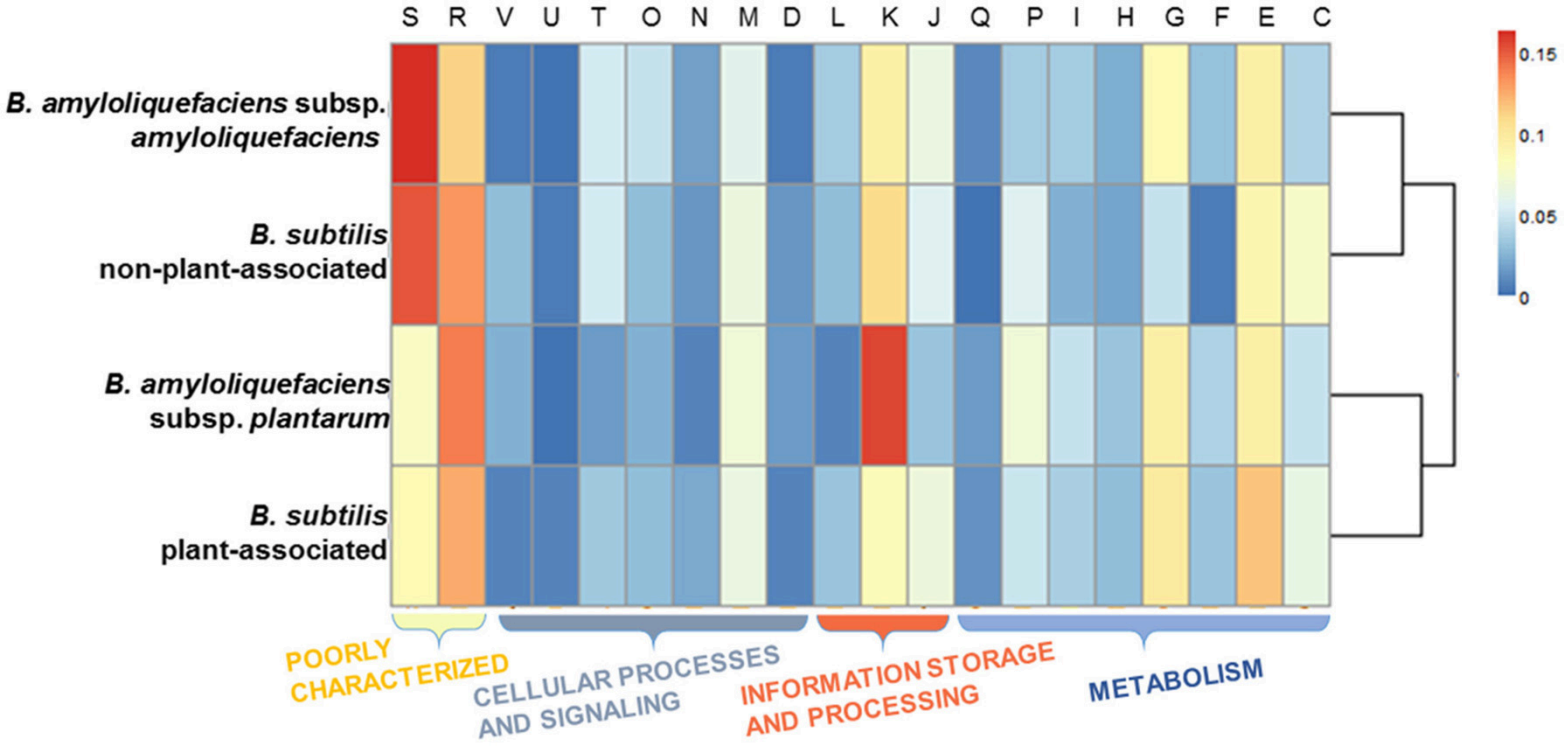
**Heat map representing the COG categories across the *four* HGT gene pools acquired by BA-nPA (non-plant-associated *B. amyloliquefaciens*), BS-nPA (non-plant-associated *B. subtilis*), BA-PA (plant-associated *B. amyloliquefaciens*), and BS-PA (plant-associated *B. subtilis*)**. The dendrogram was constructed using the pairwise Pearson's *r* correlation calculated for the protein cluster membership distributions. The cell color in the heatmap represents the percentage of gene numbers of each COG category, as revealed by the legend.

The genes were taken from the divergence gene pool of PA/nPA in both species and then assigned into COGs (Figure 4; Tables S8–S11). BS-PA acquired most genes (total 688, and 537 with COG assignment), followed by BA-nPA (233/355), BS-nPA (212/286), and BA-PA (129/192). Clustering of the PA and nPA species suggested that adaptation of both *B. subtilis* and *B. amyloliquefaciens* revealed the same tendency (Figure 5). A Venn diagram based on the four HGT pools was also generated but no obvious trends for gene distribution could be observed (Figure S1). Genes acquired at the divergence event by the four groups (BA-PA vs. BA-nPA, and BS-PA vs. BS-nPA) mainly belonged to categories E (amino acid transport and metabolism, 9.0–11.8%), G (carbohydrate transport and metabolism, 4.7–9.9%), and K (transcription, 8.4–15.6%), implying that new features in intermediary metabolism and transcriptional regulation were important events during evolution under different environmental conditions. Further, the BA-PA acquired relatively more genes in categories C (energy production and conversion, 4.7 vs. 3.9%), G (9.3 vs. 8.6%), and K (15.5 vs. 9.4%), than did in BA-nPA. Genes in categories E (11.7 vs. 9.0%), G (9.9 vs. 4.7%), and N (cell motility, genes involved in flagellum synthesis and chemotaxis, 2.0 vs. 1.4%) were over-represented in BS-PA compared with BS-nPA (Tables S8–S11). Additionally, the antibiotic resistance genes (ARGs) in the four HGT pools acquired by BA-PA, BA-nPA, BS-PA, and BA-nPA were identified using the Resfams HMM profiles (E-value: 1e-5), and 87, 131, 312, and 100 ARGs were detected in the four pools, respectively (Tables S12–S15). Numerous ARGs belonging to various types could be detected, including those encoding major facilitator superfamily (MFS)-type transporter, acetyltransferase, glyoxalase, β-lactamase, ABC transporter/efflux, and so on (Tables S12–S15). Interestingly, the proportion of ARGs to total HGT genes in BA-PA (Table S12; 87/192, 45.3%) and BS-PA (Table S14, 312/688, 45.3%) was higher than that in BA-nPA (Table S13; 131/355, 36.9%) and BS-nPA (Table S15; 100/286, 35.0%), suggesting that the plant-associated strains preferred to acquiring ARGs as compared with non-plant-associated strains at the divergence event.

Only a few genes were shared by the PA-specific core genome and the group of genes acquired by HGT at the divergence event. Of the 192 genes acquired by the BA-PA, only 18 genes (9.4%) were also present in the PA-specific core genome, including *mtlA* (GP102111, mannitol transporter) and *glnQ* (GP102791, glutamine transporter) that might be relevant to rhizosphere adaptation (Table S8). For BS-PA, the overlap of genes between HGT and specific core genome was found to be 53 of 688 (7.7%), which included *bglC*/*eglS* (GP103033, cellulase C) and *glnM* (GP102793, glutamine ABC transporter) (Table S10).

We then pay attention to the last question raised in the introduction: What's the evolutionary process of the 96 PA-specific genes (56 BA-PA specific genes and 40 BS-PA specific genes, Tables S5, S6), did they were absent in the ancestor but were acquired by the PA strains during the history or were shared by all strains but were subsequently discarded by the nPA strains? Analysis based on the AnGST model indicated that all the PA-specific genes were absent in both ancestors of *B. amyloliquefaciens* or *B. subtilis*, and only nine genes (including *bglC*/*eglS*) were predicted to be obtained by all PA strains at the initial divergence event via HGT, being BA-PA or BS-PA branches (Table S16). Most of the remaining PA-specific genes were primarily acquired by subgroups of the PA strains (probably from outgroup organisms) at the divergence event (BA-PA or BS-PA), and were then introduced to other strains through HGT during the following evolutionary process (Data Sheet S2). In detail, taking gene *mlnH* (polyketide macrolactin synthase, GP103539) for example, it firstly occurred in the ancestor of group “Y2-YAB9601-NAUB3-CC178-FZB42-AS43-UCMB5033-UCMB5113-UCMB5036-SQR9,” thereafter it was transferred from the ancestor of group “CC178-FZB42-AS43-UCMB5033-UCMB5113-UCMB5036” to the ancestor of group “IT45-CAB946,” followed by another HGT from IT45 to the ancestor of group “LFB112-NJN6” (Data Sheet S2). Evolutionary history of other PA-specific genes revealed similar patterns with that of *mlnH*, suggesting that they were not obtained by a single divergence event between PA and nPA strains, but were obtained successively by different subgroups of the PA stains during the evolution process (Data Sheet S2).

Finally, a variety of acquired genes relevant to rhizosphere adaptation at the divergence event, including those involved in polysaccharide utilization (Beauregard et al., 2013), antibiotics production (Ongena and Jacques, 2008), and stress resistance (Raaijmakers et al., 2008), were also identified. The HGT gene pools of BA-PA branch included NJN-6|AW02_009760 (GP100108) encoding polysaccharide deacetylase, IT-45|451347442 (GP102036) encoding arabinogalactan endo-1, 4-beta-galactosidase, and two genes involved in stress tolerance [AS 43.3|429503728 (GP102479) encoding an antibiotic transport system ATP-binding protein and *yusP* (UCMB5036|452856902, GP100085) encoding a putative multidrug-efflux transporter] (Table S8). In the BS-PA branch, genes involved in plant polysaccharides degradation [BAB-1|472330442 (GP102774) and BAB-1|472331232 (GP102331) both encoding endo-β-1, 4-xylanase, and *bglC*/*eglS* (XF-1|449094502, GP103033)], antibiotic biosynthesis [*srfAD* (XF-1|449093047, GP100275) and BAB-1|472330387 (GP100001) participate in the synthesis of lipopeptide surfactin, and plipastatin, respectively], and antibiotic resistance [BAB-1|472328995 (GP103444) encoding glyoxalase/bleomycin resistance protein/dioxygenase, and HJ5|AW03_002620 (GP100347) encoding a drug-export protein], were all predicted to have been acquired by HGT (Table S10).

## Discussion

### Factors driving the divergent evolution of PA and nPA strains

Genomic and evolutionary characteristics of *B. amyloliquefaciens* and *B. subtilis*, especially the root-colonizing strains, are gaining great interest right at present because of their potential in agriculture. In this study, we performed a comparative genomics analysis based on 29 previously published *B. amyloliquefaciens* and *B. subtilis* genomes, and two newly sequenced rhizospheric strain *B. amyloliquefaciens* NJN-6 and *B. subtilis* HJ5. The phylogeny based on core genomes indicated that strains isolated from the rhizosphere (PA strains) are distinguished from the nPA strains, the hierarchal clustering of OGs based on the presence/absence matrix in both *B. amyloliquefaciens* and *B. subtilis* revealed the similar pattern (Figure 2). These findings generally suggests that important changes in the genome of these *Bacillus* strains have occurred during adaptation to different habitats; more precisely a co-evolution with plants could be a driving factor (Hartmann et al., 2008). This implies that a specific niche, such as plant-associated habitats, influences HGT [plant-associated habitats, e.g., rhizosphere, are often conducive to HGT processes (van Elsas et al., 2003)] and highlights the consistency between the molecular clock mutation (nucleotide sequence, species phylogenetic tree) and gene gain/loss (hierarchal clustering) (Hao and Golding, 2006), which is in accordance with data demonstrating HGT among numerous *Streptococcus* species within a shared bovine environment (Richards et al., 2014).

In both trees the differentiation within *B. amyloliquefaciens* is quite clear and consistent with their habitats (Figure 2A), while in *B. subtilis* the clustering in the two trees is different and not fully coincided with their origins (Figure 2B). Since *B. subtilis* 168, PY79, ATCC 6051, and QB928 were all derived from root-colonizing strain NCIB3610 (Zeigler et al., 2008; Yu et al., 2012), it seemed that they should be clustered with the PA strains in the phylogenetic tree, as well as BSn5 isolated from calli tissue. The reason for this contradiction is still unclear: For the four domesticated strains, the subsequent mutation in laboratory might lead to differentiations, but actually the extent of genetic changes caused by domestication appears to be limited; for BSn5, maybe bacterium isolated from a given environment does not guarantee it has adapted to that particular habitat. For the clustering relationship of *B. subtilis* observed in the hierarchal clustering based on the matrix agrees, the three PA strain and four domesticated strains as well as BSn5 were clustered in one branch, but BSP1 that isolated from poultry was also included (Figure 2B). In general, the two trees for *Bacillus* still suggested that the three most representative plant-relevant strains (HJ5, BAB-1, and XF-1) could be distinguished from other *B. subtilis* strains, but further investigation including newly sequenced wild strains and eliminating the lab-domesticated strains is importantly needed for exploring the genomic characteristics between PA and nPA isolates in a more comprehensive perspective.

### PA strains possess more abundant genes involved in intermediary metabolism and antibiotics production as compared to PA strains

As the existence of root exudates, the rhizosphere represents a highly dynamic front for interactions and competitions between various organisms; microbes within this niche have to contend with each other for resources, through nutrient/space competition and direct suppression by “weapons” such as antibiotics and extracellular hydrolase (Bais et al., 2006). Comparison of the core genome between PA and nPA strains based on COGs analysis revealed that the genes involved in intermediary metabolism (categories E and G) and biosynthesis of secondary metabolites (Q) were more abundant in PA strains (Figure 3). Alcaraz et al. reported that *B. subtilis* inhabiting soil harbors a higher proportion of genes related to carbohydrate transport and metabolism (G) when compared to other *Bacillus* spp. (Alcaraz et al., 2010). Barret et al. also reviewed the bacterial genes expressed during PGPR-plant interactions in the rhizosphere that reported by previous studies, and indicated that the genes relevant to rhizosphere competence were mainly involved in central metabolism, detoxification and stress, and secretion (Barret et al., 2011). Therefore, the presence of the genes mentioned above could have the advantageous results of increasing the capacity to utilize various plant-derived substrates (e.g., polysaccharides as main components of plant cell wall and amino acids in root exudates) and synthesizing antibiotics, which would contribute to competitive edge and rhizosphere adaptation (Borriss et al., 2011).

Noticing that some genes identified in PA-specific core-genome are only absent in one or a few nPA strains but still exist in most of the nPA strains, a further PA-specific gene analysis was performed for exploring the categorical genomic differences between PA and nPA strains. Several genes involved in plant-derived polysaccharides degradation (*bglC*/*eglS* and *amyE*) and antibiotics production (*dfn* and *mln* clusters) were identified specifically from BA-PA strains (Table S5), suggesting that these two groups of genes are probably the most important characteristic that contribute to rhizosphere adaption of PA strains and their differences from nPA stains (Borriss et al., 2011; Wu et al., 2015). However, the BS-PA specific genes revealed limited relationship with rhizosphere adaptation, maybe further analysis based on more wild type *Bacillus* genomes could provide significant information.

### Successively HGT of rhizospheric-specific genes acquired by different subgroups of PA strains

HGT is recognized as an important factor that drives evolution and differentiation of different organisms. Itakura et al., demonstrated that the *Bradyrhizobium japonicum* strains acquired additional DNA fragments (e.g., genomic islands) through HGT performed superior symbiotic nitrogen-fixation capability than other relative strains (Itakura et al., 2009). Recently, Richards et al. investigated the dynamics of genome evolution of *Streptococcus* spp. by using AnGST, and found that eight GO groups were acquired during the early evolution/divergence events, thus enabling a better adaptation to new habitats (Richards et al., 2014). In this study, we performed an AnGST analysis to explore the gene gain/lost events during the evolutionary process of the two subspecies of *Bacillus* spp. (Figure 4). It was found that the major genes acquired by both BA-PA and BS-PA are involved in metabolism (categories E and G), transcription/signal transduction (K or KT), and synthesis of secondary metabolites (Q) (Tables S8, S10), which are all potentially important for rhizosphere survival and competition (Alcaraz et al., 2010). Especially, the higher proportion of ARGs in the HGT pools of PA strains than nPA strains at the divergence event (Tables S12–S15) suggested their roles in adaptation to various antibiotic stress in the rhizosphere.

Interestingly, only a few of the PA-specific genes could be recovered from the HGT pools of BA-PA and BS-PA branches (Tables S8, S10, S12), while most of them were acquired by subgroups of PA strains at the initial divergence event, and then transferred to other groups of PA strains successively (Data Sheet S2). This finding suggested that the rhizosphere-specific genes were not obtained by a single event, but rather were acquired by members of subpopulation that might have first migrated to the rhizosphere. Transfer of these genes to subsequently arising populations, and maintenance of acquired genes resulted in the contemporary genomic differences currently observed between PA and nPA. This phenomenon provides new information for better understanding the evolutional process of *Bacillus* spp. isolated from different niches.

### Transcriptional profiling in response to maize root exudates

Recently, the transcriptome analysis of *B. amyloliquefaciens* SQR9 was reported (a member of the BA-PA that also isolated by our laboratory) incubated with maize root exudates [which partially mimicked the presence of a rhizosphere (Zhang et al., 2015)]. Thirteen genes that belong to PA-specific core genome and were gained through HGT at the divergence event between PA and nPA (BA-PA branch), were observed to be induced by root exudates (Tables S3, S8, S10), including *mtlA* (GP102111, mannitol transporter) and *glnQ/M* (GP102791/GP102793, glutamine transporters) involved in transport of important compounds in root exudates (Badri and Vivanco, 2009). Another significant gene was *ycbA* that encodes a transcriptional regulator involved in biofilm formation (GP102097) (Stanley et al., 2003) (Tables S3, S8, S10).

Additionally, partial genes in PA-specific core genome or in the BA-PA/BS-PA HGT group also revealed positive response to root exudates, including those involved in plant-derived carbohydrates transport and metabolism, cell motility and chemotaxis, biofilm formation, and transcriptional regulation (Tables S3, S8, S10). Unfortunately, most of the significant genes belong to the BA-PA specific genes were identified as hypothetical proteins (Tables S3, S8, S10). In general, the data from the transcriptomic analysis implicated the genes in the PA-specific core genome and HGT-derived pool could be involved in rhizospheric interactions, which suggests that genes were specifically acquired by the plant-colonizing *Bacillus* spp. strains.

## Conclusion

Comparative genomic analysis of *B. amyloliquefaciens* and *B. subtilis* indicated that the plant-associated (PA) and non-plant-associated (nPA) strains were clearly distinguished. Genes relevant to plant-derived polysaccharides utilization and antibiotics synthesis are more abundant in the core genome of PA strains. Only a few of these rhizospheric-specific genes were acquired by PA strains at the initial divergence event, but most were obtained successively by different subgroups of PA stains. Our study offers new information regarding genomic differences between PA and nPA *B. subtilis* and *B. amyloliquefaciens* from different habitats and the relevant evolutionary traits, and has implications for screening of PGPR strains for application in agriculture production.

## Materials and methods

### Strains used in this study

Two newly isolated rhizospheric strains, *B. amyloliquefaciens*NJN-6 (from the banana rhizosphere) and *B. subtilis* HJ5 (from the cotton rhizosphere), were used in this study. They are both PGPR strains with outstanding plant growth promotion and biocontrol performance (such as suppression of *Fusarium* wilt of banana and *Verticillium* wilt of cotton, respectively) (Li et al., 2013; Yuan et al., 2013). NJN-6 produces several antagonistic compounds, including homolog of the cyclic lipopeptide iturin A (bacillomycin D, macrolactin A/E) and numerous volatile compounds with antifungal activity (Yuan et al., 2011, 2012a,b, 2014). HJ5 colonizes cotton roots and forms a robust biofilm (Li et al., 2013). These two strains were then selected for whole genome sequencing to obtain the relevant molecular genetic information.

### Genome sequencing and annotation

The genome sequences were obtained using shotgun sequencing with a combination of Sanger sequencing and 454 FLX system with 28 × coverage and 30.5 × coverages for *B. amyloliquefaciens* NJN-6 and *B. subtilis* HJ5, respectively. Assemblies were performed using Newbler, version 2.3 (Margulies et al., 2005) resulting in 53 and 50 contigs for NJN-6 and HJ5, respectively. Gaps in the assembly and regions where the sequence was uncertain were completed by Sanger sequencing. Predictions of protein-coding genes were implemented in Prodigal (Hyatt et al., 2010). Functional annotation was carried out using BLASTP, with *B. amyloliquefaciens* subsp. *plantarum* FZB42^T^ and *B. subtilis* 168 as references for NJN-6 and HJ5, respectively, and against GenBank's non-redundant protein databases (nr) with an E value of <1e-5. Protein clusters and domains were assigned by an RPS-BLAST search against the COG (Tatusov et al., 2003) and PFAM databases (Punta et al., 2012), with an E value of <1e-5. Transfer RNA genes (tRNA) and rRNA were identified separately using tRNAscan-SE (Lowe and Eddy, 1997) and RNAmmer 1.2 server (Lagesen et al., 2007).

### Reference genomes and identification of gene orthologous groups

Twenty nine complete genome sequences, and associated protein functions, for *B. amyloliquefaciens* and *B. subtilis* were downloaded from NCBI (12th June 2013, ftp://ftp.ncbi.nih.gov/genomes/Bacteria/).

Orthologous groups were delimited using OrthoMCL version 2.9 (Li et al., 2003), in which all the proteins sequences were compared using a BLASTP all-against-all search with an E value cutoff of <1e-05 and percent match cutoff of > 70%. Then, the Markov Cluster (MCL) algorithm was used to delineate proteins into orthologous groups (OGs) using an inflation value of 1.8 (Enright et al., 2002), which is robust to fluctuations of inflation (Brohee and van Helden, 2006). The putative pan-genome, core genome and lineage-specific gene sets were extracted from the OrthoMCL output.

### Phylogenomic analyses

A phylogenetic tree for the set of 31 genomes from the two species was inferred using a core genome alignment concatenation approach. 1836 OGs, which were present in all genomes as single-copy genes, were considered as phylogenetic markers. The multiple sequence alignments were constructed in the AUQA software (Muller et al., 2010), which relies on MUSCLE (Edgar, 2004) and MAFFT (Katoh and Standley, 2013) as aligners. The resulting 1836 amino acid alignments were combined, which resulted in a 551,710 amino acid concatenated dataset. The Gblocks server with defaults settings (Castresana, 2000) was used to remove non-alignable regions resulting in 500,457 amino acids final alignment. A maximum likelihood tree was built with IQ-TREE (version 1.1.0) (Minh et al., 2013) using the LG+I+G4+F substitution model (Le and Gascuel, 2008), and a consensus tree was constructed from 10,000 bootstrap trees. A Bayesian phylogram was obtained in MrBayes 3.2.1. (http://mrbayes.sourceforge.net/) run on CIPRES server (https://www.phylo.org/) with 200,000 mcmc generations (maximum achievable for such a large dataset); every 100 trees were sampled and a consensus tree was obtained after the first 100 generations were removed with burnin command. Gene phylogenetic trees were constructed using OGs containing three or more genes in IQ-TREE (Minh et al., 2013) with the GTR + I + G substitution model and 1000 bootstrap replicates.

### Hierarchal clustering

To evaluate the uncertainty of speciation from the OGs that were not shared among all the genomes analyzed in this study, we first constructed a 0/1 matrix with 1 indicating that the OG was present in a genome and 0 indicating the absence of that OG. OGs that were only shared by partial strains were probably obtained through HGT (Zhu et al., 2014). The hierarchical clustering was used to analyze the 0/1 matrix as implemented in the R package pvclust (Suzuki and Shimodaira, 2006) with 500 bootstrap replicates.

### Reconciliation between species tree and gene tree

A parsimony-based reconciliation approach, AnGST, was used to understand the history of the PA genomic evolution events (David and Alm, 2011). The reconciliation was obtained by inferring a minimum set of evolutionary events between the gene- and species-trees, including horizontal gene transfer (HGT), gene duplication (DUP), gene loss (LOS), speciation (SPC), and one gene birth or genesis event (GEN). All the OGs composed of more than two proteins were analyzed by AnGST using the species tree and default parameters. Inference errors due to phylogenetic uncertainty were minimized by incorporating 1000 bootstrap replicates per gene. For those OGs containing only two genes, one of the genes was observed once in two separate taxa or twice in one taxa. For these genes, we followed the methods of Richards (Richards et al., 2014) to overlay the position of the two genes onto the species tree, using the same set of evolutionary events and event penalties as described above.

The antibiotic resistance genes in the four HGT pools acquired by BS-PA, BS-nPA, BA-nPA, BA-PA were identified by searching against ResFams (v1.2) (Gibson et al., 2015) using HMMER (v3.1b2; E-value: 1e-5) (Eddy, 2011). Venn diagram based on the four gene pools was generated with R package VennDiagram (Chen and Boutros, 2011).

## Author contributions

Conceived and designed experiments: ID, CK, QS, and RZ. Performed the experiments: NZ. Performed bioinformatic analysis: DY and JK. Wrote the paper: NZ, DY, RB, ID, and RZ. All authors read and approved the final manuscript.

### Conflict of interest statement

The authors declare that the research was conducted in the absence of any commercial or financial relationships that could be construed as a potential conflict of interest.

## Supplementary material

Table S1 **Statistics for the core-genome and pan-genome of the 31 Bacillus strains**.

Table S2 **Groups divided into plant-associated (PA) and non-plant-associated (nPA) based on the species phylogenetic tree**.

Table S3 **Gene products and COG assignments of the PA-specific core genome**. The genes that showed homology with SQR9's were appended with the different gene expression patterns regulated by root exudate at 24 and 48 h post-inoculation.

Table S4 **Gene products and COG assignments of the nPA-specific core genome**. The genes that showed homology with SQR9's were appended with the different gene expression patterns regulated by root exudate at 24 and 48 h post-inoculation.

Table S5 **Genes that existed in all *Bacillus amyloliquefaciens* subsp. *plantarum* (BA-PA) but not in *Bacillus amyloliquefaciens* subsp. *amyloliquefaciens* (BA-nPA)**.

Table S6 **Genes that existed in *Bacillus subtilis* plant-associated (BS-PA) but not in *Bacillus subtilis* non-plant-associated (BS-nPA)**.

Table S7 **Statistics of evolutionary events derived from AnGST along each branch. hgt (total), total gene count of genes involved in horizontal gene transfer, speciation and gene duplication**. spc, speciation; dup, gene duplication; los, gene loss; brn, gene birth; cur, gene counts in current strain.

Table S8 **Gene products and COG assignments of predicted HGT in *Bacillus amyloliquefaciens* subsp. *plantarum* (BA-PA)**. The genes that showed homology with SQR9's were appended with the different gene expression patterns regulated by root exudate at 24 and 48 h post-inoculation.

Table S9 **Gene product and COG assignments of predicted HGT in *B. amyloliquefaciens* subsp. *amyloliquefaciens* (BA-nPA)**. The genes that showed homology with SQR9's were appended with the different gene expression patterns regulated by root exudate at 24 and 48 h post-inoculation.

Table S10 **Gene products and COG assignments of predicted HGT in *B. subtilis* plant-associated (BS-PA)**. The genes that showed homology with SQR9's were appended with the different gene expression patterns regulated by root exudate at 24 and 48 h post-inoculation.

Table S11 **Gene products and COG assignments of predicted HGT in *B. subtilis* non-plant-associated (BS-nPA)**. The genes that showed homology with SQR9's were appended with the different gene expression patterns regulated by root exudate at 24 and 48 h post-inoculation.

Table S12 **Antibiotic resistance genes (ARGs) of HGT on the plant-associated *Bacillus amyloliquefaciens* (BA-PA) predicated by ResFams**.

Table S13 **Antibiotic resistance genes (ARGs) of HGT on the non-plant-associated *Bacillus amyloliquefaciens* (BA-nPA) predicated by ResFams**.

Table S14 **Antibiotic resistance genes (ARGs) of HGT on the plant-associated *Bacillus subtilis* (BS-PA) predicated by ResFams**.

Table S15 **Antibiotic resistance genes (ARGs) of HGT on the non-plant-associated *Bacillus subtilis* (BS-nPA) predicated by ResFams**.

Table S16 **List of genes that were BA/BS-PA specific and were involved in HGT at the divergence event of PA and nPA**.

Figure S1 **Venn diagram based on the four HGT gene pools acquired by BA-PA (plant-associated *B. amyloliquefaciens*), BS-PA (plant-associated *B. subtilis*), BS-nPA (non-plant-associated *B. subtilis*), and BA-nPA (non-plant-associated *B. amyloliquefaciens*)**.

Data Sheet 1 **Orthologous groups derived from OrthoMCL**.

Data Sheet 2 **The evolutionary history of each gene copy for genes related to rhizosphere adaptation**.
