# Supplementary figures and images for: Comparative Genomic Analysis of Bacillus amyloliquefaciens and Bacillus subtilis Reveals Evolutional Traits for Adaptation to Plant-Associated Habitats
Source: Front Microbiol. 2016 Dec 20;7:2039. doi: 10.3389/fmicb.2016.02039 (PMC5169363; doi:10.3389/fmicb.2016.02039)

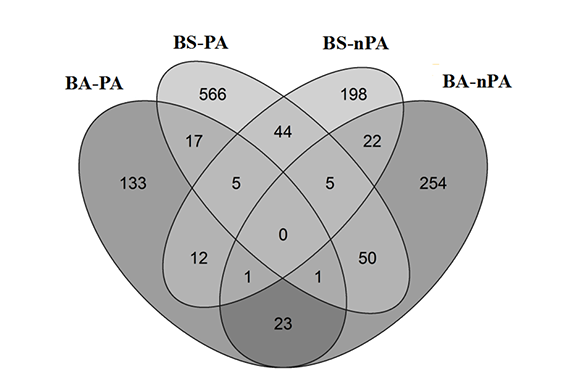

Supplement: Figure S1 — Venn diagram based on the four HGT gene pools acquired by BA-PA (plant-associated B. amyloliquefaciens), BS-PA (plant-associated B. subtilis), BS-nPA (non-plant-associated B. subtilis), and BA-nPA (non-plant-associated B. amyloliquefaciens). [file Image1.TIF]
